# Validation of the food allergy quality‐of‐life questionnaire—Parent form for adolescents (FAQLQ‐PFA)

**DOI:** 10.1111/pai.70534

**Published:** 2026-10-23

**Authors:** Rebecca C. Knibb

**Affiliations:** ^1^ Institute of Health and Neurodevelopment, Aston University Birmingham UK

**Keywords:** adolescents, caregivers, food allergy quality of life, measurement, teens

To the Editor,

The impact of food allergy (FA) on quality‐of‐life (QoL) in adolescent patients is well established.^1^ Research has consistently shown an association with poorer social QoL, including social situations involving food and social networks^1^ and with emotional QoL, where anxiety about an accidental reaction is particularly prevalent.^1^ For the last 15 years we have had a suite of patient reported outcome measures for FA‐QoL, the Food Allergy Quality of Life Questionnaires (FAQLQs).^2^ These have been extensively used in research, clinical trials and clinical practice.^2^ The FAQLQ parent form for adolescents (FAQLQ‐PFA) was developed by the current author (RK) in 2011 alongside the other FAQLQs. It is distinct from the FAQLQ teenager form and is not just a parent‐proxy but was developed to directly measure parent's views of their adolescent's QoL.^3^ It is therefore different to parent‐proxy measures that simply re‐word a child‐report version so that it can be answered by the parent. Parent perceptions are important to understand as these can have an impact on children's own perceptions but can also be different to the perceptions of the child or adolescent themselves.^3^ The details of the initial development and psychometric properties of the FAQLQ‐PFA have not been published to date. Here data is presented on the original development by the author and further indices of reliability and validity of the tool in participants from the UK.

For the original scale development, ethical approval was given by the University of Derby School of Psychology Research Ethics Committee; written assent from adolescents and consent from caregivers was obtained. Semi‐structured interviews were carried out with 11 mothers, 6 fathers and 8 adolescents (aged 13–17 years) with a medical diagnosis of FA, who responded to local newspaper advertising or an advert posted by Anaphylaxis Campaign (now Anaphylaxis UK). Interviews were audio recorded, transcribed verbatim and content analysis was used to extract items for a prototype scale, which had 113 items, scored on a scale of 1–7 with a higher score representing a bigger impact or poorer QoL. This was sent out to 200 caregivers of adolescents with a medical diagnosis of FA, by post by the Anaphylaxis Campaign; 87 completed scales were returned. Following methodology used for the other FAQLQs,^2^ a clinical impact score was calculated for each item (proportion of participants saying an item had an impact multiplied by the mean rating for that item). Items with a clinical impact score of >2.0 were retained (*n* = 24). Four of these items were discarded as they overlapped significantly and six further items were included based on clinical experience of what was important to retain, giving a total of 26 items.

The 26‐item scale was sent out by post by the Anaphylaxis Campaign to a further 200 caregivers, alongside the Child Health Questionnaire (CHQ‐PF50)^4^ and the Food Allergy Independent Measure (FAIM).^5^ A total of 66 caregivers completed all scales in full; 67% were caregivers for male adolescents, mean age of adolescent was 14.84 years (range 13–17, S.D. = 1.42), 91% allergic to peanut, 70% allergic to tree nuts. Principal Components Analysis (PCA) with varimax rotation and eigenvalues >1 was run to explore the factor structure of the scale. The KMO was .86 and Bartlett's *χ* ^2^ = 1091.04, *p* < .001 indicating the sample was appropriate to run factor analysis. The initial solution was a 6‐factor structure which was not very interpretable. However, the loadings for the 6‐factor structure and scree plot suggested a 4‐factor structure may be optimal and so this was run. This resulted in an interpretable structure, explaining 62.68% of the variance. Factors were called Dietary Frustrations, Social Restrictions, Psychological Impact and Food Allergy Awareness (Table 1).

**TABLE 1:** Principle components analysis of the items of the FAQLQ‐PFA.

	Factor 1	Factor 2	Factor 3	Factor 4
Factor 1: Dietary Frustrations (alpha 0.91)				
My teenager misses out because of food allergy	**0.782**			
My teenager gets frustrated because of food allergy	**0.755**			
My teenager has a restricted diet because of food allergy	**0.748**			
My teenager gets irritated by his/her food allergy	**0.688**	0.534		
My teenager has to be more responsible than other teenagers because of food allergy	**0.652**			
My teenager always eats the same foods because of food allergy	**0.600**			
My teenager cannot experiment with different foods on holiday because of food allergy	**0.531**			
My teenager feels different to other teenagers because of food allergy	**0.520**			
My teenager feels anxious in restaurants	**0.519**			
Factor 2: Social Restrictions (alpha 0.88)				
My teenager finds it difficult to ask about food ingredients in restaurants		**0.829**		
My teenager avoids telling people about his/her food allergy until he/she knows them well		**0.786**		
My teenager worries as he/she always has to carry a bag because of his/her medication		**0.556**		
My teenager worries that he/she can only eat in a limited range of restaurants	0.498	**0.529**		
My teenager feels different because she/he can't eat what their friends can eat	0.444	**0.479**		
My teenager sticks to foods he/she knows	0.403	**0.459**	0.493	
School trips away are not as easy for my teenager because of food allergy		**0.451**		0.431
Factor 3: Psychological Impact (alpha 0.78)				
My teenager has been really scared by having a reaction			**0.770**	
My teenager gets frightened about food allergy			**0.623**	
My teenager feels nervous around the food they are allergic to because of food allergy			**0.618**	
I feel my teenager has had to grow up more quickly because of food allergy	0.433		**0.521**	
My teenager has been teased because of food allergy			**0.449**	
My teenager is more cautious generally because of food allergy	0.544		**0.465**	
Factor 4: Food Allergy Awareness (alpha 0.82)				
My teenager takes more of an interest in food because of food allergy				**0.850**
My teenager reads the label on everything he/she eats				**0.827**
My teenager is frustrated about food labelling				**0.621**
My teenager is more wary of situations because of food allergy			0.407	**0.552**
Eigenvalues	**5.54**	**3.90**	**3.48**	**3.38**
Variance	**21.29**	**14.99**	**13.40**	**13.00**

*Note*: All factor loadings above 0.4 are reported; 2 items were placed in a factor that was a better conceptual fit rather than the factor that related to the highest factor loading. Values in bold denote the factor the item was placed on.

Cronbach's alpha for the full scale (.90) and for the sub‐scales (.78–.91) was good to excellent (Table 1). Poorer QoL as measured by FAQLQ‐PFA significantly correlated with poorer CHQ‐PF50 total score and sub‐scales for global health, health perception, family activities and impact on parental time, demonstrating good cross‐sectional convergent validity (Table 2). FAQLQ‐PFA total scores and all sub‐scale scores significantly correlated with the FAIM, demonstrating construct validity. Poorer QoL also correlated with more reported FA symptoms (see Table 2).

**TABLE 2:** Means scores for the FAQLQ‐PFA and correlations between the FAQLQ‐PFA, CHQ‐PF50, FAIM and symptoms.

	FAQLQ‐PFA	DF	SR	PI	FAA
Mean (SD) scores	4.02 (1.32)	4.14 (1.47)	3.73 (1.66)	3.79 (1.35)	4.45 (1.62)
CHQ‐PF50					
CHQ‐PF50 total	−.32**	−.40**	−.27*	−.19	−.17
Family activities	−.32**	−.35**	−.24*	−.21	−.27*
Mental health	−.19	−.19	−.21	−.16	−.02
Role social emotional	−.04	−.11	.01	.02	.01
Global health	−.30*	−.33**	−.25*	−.28*	−.11
Physical functioning	−.16	−.21	−.04	−.15	−.16
Role social physical	−.02	−.02	.08	−.09	−.09
Bodily pain	.06	.10	.08	.01	−.07
Behavior	−.15	−.18	−.19	−.002	−.06
Global behavior	−.05	−.09	−.17	.16	.01
Self‐esteem	−.16	−.23	−.20	−.03	.06
Health perception	−.30*	−.31*	−.17	−.31*	−.27*
Change in health	−.07	−.03	−.004	−.16	−.10
Parental impact emotional	−.20	−.26*	−.16	−.12	−.14
Parental impact on time	−.30*	−.33**	−.26*	−.19	−.20
Family cohesion	−.16	−.25*	−.14	−.05	−.02
FAIM score	.47**	.47**	.34**	.51**	.30**
Number of symptoms	.29*	.25*	.19	.36**	.21

Abbreviations: DF, Dietary Frustrations; FAA, Food Allergy Awareness; PI, Psychological Impact; SR, Social Restrictions.

* *p* < .05.

** *p* < .01.

As part of the Food Sensitive study on QoL of children, adults and caregivers with food hypersensitivity across the UK run in 2020 and 2021,^6^ data on the FAQLQ‐PFA was collected in two waves, 1 year apart. Caregivers of adolescents with medically diagnosed FA responded to adverts by patient organizations or to an advert to a survey panel run by Qualtrics. They completed the scale online, alongside a generic measure of QoL (EQ‐5D‐Y parent proxy).^7^ Written consent was obtained from caregivers and ethical approval was given by the Aston University Research Ethics Committee.

A total of 78 caregivers completed the scales in wave 1 of the Food Sensitive study and 83 in wave 2. In wave one, 68% of caregivers were female, 83% white British, mean age of adolescent was 14.67 years (range 13–17, SD = 1.37), 34.6% female, 46% allergic to peanut, 50% allergic to tree nuts, and 20% to eggs. In wave two, 71% of caregivers were female, 85.5% white British, mean age of adolescent was 14.59 (range 13–17 years, SD = 1.42), 44.6% female, 42% allergic to peanut, 45% to tree nuts, and 33% to eggs.

In both Food Sensitive study samples, the FAQLQ‐PFA demonstrated excellent internal reliability for the full scale (Cronbach's alpha .95–.97) and sub‐scales (.80–.93) (Table 3). Poorer QoL as measured by the FAQLQ‐PFA significantly correlated with poorer general QoL as measured by the EQ‐5D‐Y and caregiver perceived severity of adolescent's FA (Table 3). Caregivers also reported poorer QoL if their adolescent had been prescribed an adrenaline auto‐injector or experienced anaphylaxis (all *p*'s < 0.05) (see eTable S1).

**TABLE 3:** Mean scores and Cronbach's alpha for FAQLQ‐PFA and correlations between the FAQLQ‐PFA, EQ‐5D‐Y, perceived severity score and number of symptoms.

	FAQLQ‐PFA	DF	SR	PI	FAA
Wave 1 (*n* = 78)					
Cronbach's alpha	.97	.94	.93	.88	.85
Mean (SD) scores	4.61 (1.44)	4.74 (1.51)	4.48 (1.63)	4.48 (1.51)	4.73 (1.50)
Caregiver perceived severity	.51**	.53**	.50**	.40**	.40**
Number of foods allergic to	.21	.28*	.24*	.01	.10
EQ‐5D‐Y					
Mobility	.06	.08	.08	.001	.06
Looking after him/herself	−.14	−.20	−.13	−.09	−.06
Doing usual activities	.22	.24*	.17	.18	.20
Having pain or discomfort	.30**	.30**	.26*	.27*	.28*
Feeling worried, sad or unhappy	.42**	.37**	.40**	.40**	.42**
Wave 2 (*n* = 83)					
Cronbach's alpha	.95	.80	.91	.90	.86
Mean (SD) scores	4.97 (1.10)	4.76 (.95)	4.87 (1.48)	5.17 (1.43)	5.30 (1.34)
Caregiver perceived severity	.39**	.28*	.39**	.31**	.35**
Number of foods allergic to	.09	.01	.12	.01	.02
EQ‐5D‐Y					
Mobility	.22*	.13	.25*	.23*	.13
Looking after him/herself	.13	.05	.18	.16	.03
Doing usual activities	.32**	.21	.36**	.32**	.21
Having pain or discomfort	.27*	.25*	.27*	.23*	.16
Feeling worried, sad or unhappy	.32**	.23*	.28*	.32**	.27*

Abbreviations: DF, Dietary Frustrations; FAA, Food Allergy Awareness; PI, Psychological Impact; SR, Social Restrictions.

* *p* < .05.

** *p* < .01.

These results show that the FAQLQ‐PFA is a reliable and valid tool for measuring caregivers' views of QoL in their adolescents (see e‐supplement for the full FAQLQ‐PFA and scoring information). The correlations with the CHQ‐PF50 and the EQ‐5D‐Y were low to medium in strength, demonstrating the unique nature of FA‐QoL and the importance of using FA‐specific measures. The strongest correlations with the EQ‐5D‐Y were with general feelings of worry, sadness or unhappiness and pain or discomfort, rather than mobility. However, in wave two of the Food Sensitive study, correlations with usual activities show that FA can interact with all aspects of daily life. The tool was also significantly associated with indices of FA severity. These patterns of association have been found in other work on FA and QoL^1^, ^8^, ^9^ and corroborate the validity of the FAQLQ‐PFA.

Further work could be carried out on this scale to confirm the factor structure and create minimal important clinical difference scores. A test re‐test needs to be run to confirm temporal stability. Norm data would also be useful; however, the mean scores from these UK datasets suggest that for this population you would expect total FAQLQ‐PFA scores of 4 to 5 out of 7. There are some limitations to consider. Detailed demographic information on caregivers was not collected for the original developmental and validation samples, and the further validation sample was a homogenous group of largely white British females. Further work on under‐represented populations is needed, including translation into different languages and provision of the tool in different modes.

Health‐related QoL has recently been recommended as a core outcome that should be measured in any intervention for FA.^10^ The FAQLQ‐PFA has been extensively used in research and clinical trials. The data presented here provide confidence that this scale demonstrates good psychometric properties and should be used to inform future research, trial efficacy and clinical practice.

## AUTHOR CONTRIBUTIONS

**Rebecca C. Knibb:** Conceptualization; investigation; funding acquisition; writing – original draft; methodology; validation; visualization; writing – review and editing; software; formal analysis; project administration; data curation; supervision; resources.

## FUNDING INFORMATION

Food Standards Agency funded the Food Sensitive study (Project code FS430406).

## CONFLICT OF INTEREST STATEMENT

Research funding from the National Institute for Health Research, Natasha's Foundation, Aimmune, National Peanut Board, Novartis, and the Food Standards Agency, and honoraria from ALK, Nutricia, Viatris, Stallergenes Greer, and DBV Technologies. R.C.K. is also Chair of the British Society for Allergy and Clinical Immunology Psychology Special Interest Group.

## Supporting information


**eTable S1.** Comparison of FAQLQ‐PFA mean scores (standard deviations) for prescription of AAI and experience of anaphylaxis.

## Data Availability

The data that support the findings of this study are available from the corresponding author upon reasonable request.
