# Supplementary material for: Validation of the food allergy quality‐of‐life questionnaire—Parent form for adolescents (FAQLQ‐PFA)
Source: Pediatr Allergy Immunol. 2026 Oct 23;37(10):e70534. doi: 10.1111/pai.70534 (PMC13691612; doi:10.1111/pai.70534)
Supplement: Supplementary file 1 [file PAI-37-e70534-s001.docx]

Supplementary file

eTable 1. Comparison of FAQLQ-PFA mean scores (standard deviations) for prescription of AAI and experience of anaphylaxis

| **FAQLQ-PFA** | **Prescribed AAI** | | **T value** | **Anaphylaxis** | | **T value** |
| --- | --- | --- | --- | --- | --- | --- |
|  | Yes | No |  | Yes | No |  |
| **Food Sensitive wave 1** |  |  |  |  |  |  |
| FAQLQ-PFA Total | 5.12 (1.18) | 3.81 (1.46) | 4.32*** | 5.19 (1.25) | 3.85 (1.41) | 4.23*** |
| Dietary Frustrations | 5.31 (1.22) | 3.87 (1.49) | 4.62*** | 5.37 (1.26) | 3.90 (1.48) | 4.53*** |
| Social Restrictions | 5.06 (1.39) | 3.58 (1.59) | 4.30*** | 5.16 (1.50) | 3.63 (1.51) | 4.28*** |
| Psychological Impact | 4.90 (1.36) | 3.82 (1.53) | 3.23** | 4.96 (1.38) | 3.84 (1.49) | 3.30** |
| Food Allergy Awareness | 5.15 (1.35) | 4.07 (1.53) | 3.23** | 5.16 (1.44) | 4.17 (1.49) | 2.83** |
|  |  |  |  |  |  |  |
| **Food Sensitive wave 2** |  |  |  |  |  |  |
| FAQLQ-PFA Total | 5.19 (0.90) | 4.43 (1.33) | 2.63** | 5.22 (0.91) | 4.55 (1.33) | 2.28* |
| Dietary Frustrations | 4.94 (0.80) | 4.33 (1.14) | 2.41* | 4.87 (0.84) | 4.54 (1.14) | 1.31 |
| Social Restrictions | 5.17 (1.31) | 4.16 (1.64) | 2.98** | 5.18 (1.27) | 4.36 (1.67) | 2.25* |
| Psychological Impact | 5.37 (1.24) | 4.73 (1.73) | 1.67 | 5.51 (1.17) | 4.64 (1.81) | 2.19* |
| Food Allergy Awareness | 5.56 (1.01) | 4.68 (1.77) | 2.34* | 5.66 (1.01) | 4.79 (1.71) | 2.35* |

***p<0.001; **p<0.01: *p<0.05

**Food Allergy Quality of Life Questionnaire – Parent Form - Adolescents Aged 13-17**

Instructions for participants

- The following are all scenarios that parents have told us affect their adolescent’s quality of life because of food allergy.
- Please indicate how much of an impact each scenario has on your teenager’s quality of life by selecting one of the boxes number 1-7.
- If you believe the scenario has no impact please choose 1 (not at all). It is important that you answer all the questions to help us understand the impact of food allergy on the quality of life of teenagers.

|  |  | ***Response choice*** | | | | | | |
| --- | --- | --- | --- | --- | --- | --- | --- | --- |
|  | **Question** | **1** | **2** | **3** | **4** | **5** | **6** | **7** |
|  |  | Not at all | Barely | Slightly | Moderately | Quite a bit | Very much | Extremely |
|  | **Dietary Frustrations** |  |  |  |  |  |  |  |
| **1.** | My teenager misses out because of food allergy |  |  |  |  |  |  |  |
| **2.** | My teenager gets frustrated because of food allergy |  |  |  |  |  |  |  |
| **3.** | My teenager has a restricted diet because of food allergy |  |  |  |  |  |  |  |
| **4.** | My teenager gets irritated by his/her food allergy |  |  |  |  |  |  |  |
| **5.** | My teenager has to be more responsible than other teenagers |  |  |  |  |  |  |  |
| **6.** | My teenager always eats the same foods because of food allergy |  |  |  |  |  |  |  |
| **7.** | My teenager cannot experiment with different foods on holiday because of food allergy |  |  |  |  |  |  |  |
| **8.** | My teenager feels different to other teenagers because of food allergy |  |  |  |  |  |  |  |
| **9.** | My teenager feels anxious in restaurants |  |  |  |  |  |  |  |
|  | **Social Restrictions** |  |  |  |  |  |  |  |
| **10.** | My teenager finds it difficult to ask about food ingredients in restaurants |  |  |  |  |  |  |  |
| **11.** | My teenager avoids telling people about his/her food allergy until he/she knows them well |  |  |  |  |  |  |  |
| **12.** | My teenager worries as he/she always has to carry a bag because of his/her medication |  |  |  |  |  |  |  |
|  |  | ***Response choice*** | | | | | | |
|  | **Question** | **1** | **2** | **3** | **4** | **5** | **6** | **7** |
|  |  | Not at all | Barely | Slightly | Moderately | Quite a bit | Very much | Extremely |
| **13.** | My teenager worries that he/she can only eat in a limited range of restaurants |  |  |  |  |  |  |  |
| **14.** | My teenager feels different because he/she cannot eat what his/her friends can eat |  |  |  |  |  |  |  |
| **15.** | School trips away are not easy for my teenager |  |  |  |  |  |  |  |
| **16.** | My teenager sticks to foods he/she knows |  |  |  |  |  |  |  |
|  | **Psychological Impact** |  |  |  |  |  |  |  |
| **17.** | My teenager has been really scared by having a reaction |  |  |  |  |  |  |  |
| **18.** | My teenager gets frightened about food allergy |  |  |  |  |  |  |  |
| **19.** | My teenager feels nervous around the food they are allergic to because of food allergy |  |  |  |  |  |  |  |
| **20.** | I feel my teenager has had to grow up more quickly because of food allergy |  |  |  |  |  |  |  |
| **21.** | My teenager has been teased because of food allergy |  |  |  |  |  |  |  |
| **22.** | My teenager is more cautious generally because of food allergy |  |  |  |  |  |  |  |
|  | **Food Allergy Awareness** |  |  |  |  |  |  |  |
| **23.** | My teenager takes more of an interest in food because of food allergy |  |  |  |  |  |  |  |
| **24.** | My teenager reads the label on everything he/she eats |  |  |  |  |  |  |  |
| **25.** | My teenager is frustrated about food labelling |  |  |  |  |  |  |  |
| **26.** | My teenager is more wary of situations because of food allergy |  |  |  |  |  |  |  |

**Scoring information**

Total score is calculated by summing all items and dividing by the number of items in the scale, resulting in an overall quality of life score of 1-7. There are no reverse items.

For sub-scale scores, sum all items in the sub-scale and divide by the number of items in the sub-scale, resulting in sub-scale scores between 1-7.

The FAQLQ-PFA has 3 sub-scales: Dietary Frustrations (items 1-9), Social Restrictions (items 10-16), Psychological Impact (items 17-22), Food Allergy Awareness (items 23-26).
